# Aggregation of protein therapeutics enhances their immunogenicity: causes and mitigation strategies

**DOI:** 10.1039/d1cb00067e

**Published:** 2021-05-04

**Authors:** Mimmi L. E. Lundahl, Silvia Fogli, Paula E. Colavita, Eoin M. Scanlan

**Affiliations:** School of Biochemistry and Immunology, Trinity Biomedical Sciences Institute, Trinity College Dublin Dublin 2 Ireland; Glycome Biopharma, Unit 4, Joyce House, Barrack Square, Ballincollig Co Cork P31 HW35 Ireland; School of Chemistry and Trinity Biomedical Sciences Institute, Trinity College Dublin Dublin 2 Ireland eoin.scanlan@tcd.ie

## Abstract

Protein aggregation in biotherapeutics has been identified to increase immunogenicity, leading to immune-mediated adverse effects, such as severe allergic responses including anaphylaxis. The induction of anti-drug antibodies (ADAs) moreover enhances drug clearance rates, and can directly block therapeutic function. In this review, identified immune activation mechanisms triggered by protein aggregates are discussed, as well as physicochemical properties of aggregates, such as size and shape, which contribute to immunogenicity. Furthermore, factors which contribute to protein stability and aggregation are considered. Lastly, with these factors in mind, we encourage an innovative and multidisciplinary approach with regard to further research in the field, with the overall aim to avoid immunogenic aggregation in future drug development.

## Abstract 2

Protein aggregation of biotherapeutics increases their immunogenicity, leading to immune-mediated adverse effects. In this review we discuss immune activation pathways, causes of aggregation and mitigation strategies.

## Graphical abstract

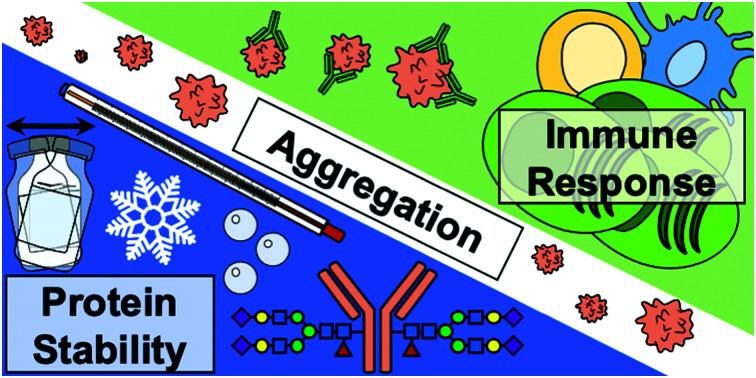

## Introduction

The use of native and native-like proteins as biotherapeutics is one of the greatest successes in the field of modern medicine. Ever since the introduction of insulin as the first therapeutic protein, it has been an ever-expanding field.^1^ Biotherapeutics are used to treat a broad range of severe diseases, for instance the immune messenger cytokines interferon beta (IFNβ) and alpha (IFNα) products are used to treat multiple sclerosis (MS)^2^ and viral diseases^3^ respectively. Moreover monoclonal antibodies are used to treat a range of diseases: autoimmune diseases – such as MS^4^ and Guillain-Barré syndrome^5^ – chronic inflammatory diseases, such as Crohn's disease,^6^ as well as numerous cancers.^7,8^ One of the major challenges of producing, distributing and storing these protein therapeutics is the risk of aggregation. Aggregation reduces the efficacy of the therapeutic by reducing its concentration and promoting its removal^9,10^ and has been shown to augment the activation of immune responses.

Protein aggregation-mediated immune activation can cause adverse side effects towards the therapeutic in question. For instance, aggregation has been linked with induction of allergic responses, including severe type 1 hypersensitivity responses, such as urticaria (wheals, sometimes accompanied by angioedema),^11,12^ or even anaphylaxis.^13,14^ Moreover, the aggregation of protein therapeutics has been shown to induce anti-drug antibodies (ADAs).^15–17^ ADAs can greatly reduce the efficacy of the therapeutic in two crucial ways. Firstly, antibodies form complexes with their target protein, and this antibody formation is a signal to immune cells to take up the complex and degrade it, which increases the clearance rate.^18–20^ Secondly, neutralizing antibodies directly impede the therapeutic function of the protein, through binding to its active site^17,21^ or preventing its function in some other manner, such as inhibiting its uptake by its cellular recipients.^22^

The production of neutralizing antibodies can have devastating effects. Development of neutralizing antibodies against IFNβ in relapse-remitting multiple sclerosis (RRMS) patients has been shown to inhibit IFNβ induced signalling^23^ and leads to an irreversible increase in disease score.^2^ Furthermore, anemic patients with chronic renal failure that were positive for neutralizing antibodies against recombinant human erythropoietin developed pure red cell aplasia (an absence of red blood cell precursors).^24^ Regarding the role of protein aggregation, in two cases of neutralizing antibodies reported during a pre-marketing clinical trial for recombinant erythropoietin, this immune response was proposed to be due to a high degree of aggregation.^25^ Aggregated human growth hormone (hGH) has moreover been associated with development of ADAs in children.^26^

In this review, we will discuss immune response to protein aggregates, focusing on activation of both innate and adaptive immunity. We will outline the major factors that can affect protein stability in a formulated environment and discuss the various approaches that have been developed for ameliorating protein aggregation. The review concludes with a discussion on the current state-of-the-art and future directions for addressing aggregation of protein therapeutics.

## The immune response

The immune response is composed of two factions: the innate and the adaptive immune response. Innate immune cells are present in essentially all tissues of the body; they detect danger, phagocytose debris, pathogens and antibody-bound peptides/microbes, as well as act as bridges for activating adaptive immune responses. The two adaptive immune cells this review will focus on are cluster of differentiation (CD)4+ T helper (Th) cells and B cells. B cells upon activation differentiate into antibody-/ADA-producing plasma cells.

T- and B cells target a specific epitope/peptide/antigen, *via* their respective T cell receptor (TCR) and B cell receptor (BCR). They become activated and differentiate upon recognition of their antigen, together with other activation signals (cell surface receptors and cytokines).^27^ Activation of antigen-specific CD4+ Th cells help activate cognate antigen-specific B cells to proliferate and become antibody producing plasma cells; a process known as T cell dependent antibody production.^28^ Antibody production can also occur *via* T cell independent means.

Certain innate immune cells, known as antigen presenting cells (APCs), are responsible for activating CD4+ T cells, by presenting antigens on the major histocompatibility complex class II (MHC II), up-regulation of other activation signals and by cytokine secretion. An important APC is the dendritic cell (DC). DCs become activated and mature by recognizing conserved molecular patterns associated with danger, *via* pattern recognition receptors (PRRs).^29^ The recognition of danger is key for the immune response to be able to distinguish between harmless and native peptides, and those associated with infection or damage. In the absence of danger, immune tolerance prevails; which involves both the absence and prevention of immune activation. In the case of immune responses against biotherapeutics which are based on native proteins, a key consideration is therefore how immune tolerance is overcome.

### Activation of innate immunity

Protein aggregation has been shown to enhance DC maturation and antigen presentation.^30–35^ For example, aggregation of trastuzumab by heat- or stir-stress enhanced the expression of co-stimulatory CD86 on human monocyte-derived DCs (MoDCs) and the stir-stressed aggregates also enhanced MHC II expression, as compared to the non-aggregated monomer formulation.^36^ Furthermore, when uptake of stir-stressed rituximab aggregates was investigated by fluorescent microscopy, it was found that the aggregated formulation was taken up by MoDCs to a greater extent than the monomer, accumulating in late endosomal compartments and enhancing MHC II presentation, as shown by marked overlap between fluorescently-labelled rituximab and MHC II receptor.^36^

Rituximab aggregation has also been demonstrated to enhance MoDC expression of maturation markers CD86 and CD83.^35^ Similarly, a combination of heat and stir-induced aggregation of two different humanized model immunoglobulin (Ig)G antibodies up-regulated MoDC expression of CD80 and CD86^31^ and stir-induced aggregation of hGH^35^ enhanced MoDC expression of maturation markers CD80, CD86, MHC II, CD40 and CD83 compared to their monomer counterparts. Aggregation of hGH, serum-purified IgG and Rituximab also enhanced MoDC secretion of the pro-inflammatory cytokine interleukin (IL)-12p40 and the chemokines CXCL10 and IL-8.^35^ Chemokines aid recruitment of more immune cells to the site of danger *via* chemotaxis: CXCL10 has been shown to recruit T cells^37^ and IL-8 propagates inflammation by recruiting innate immune cells.^38^

Regarding activation of downstream adaptive immune responses, Gallais *et al.* used aggregates of hGH, rituximab and a model IgG to see whether they could induce T cell proliferation in a MoDC co-culture.^35^ Indeed, the aggregated formulations of all three proteins induced T cell proliferation, whilst their monomeric counterparts induced no such activation. Similar studies using peripheral blood mononuclear cells (PBMCs), investigating aggregate-containing formulation of trastuzumab^36^ and a model human IgG^32^ observed enhanced CD4+ T cell proliferation, compared to each corresponding monomer formulation.

**Fig. 1:**
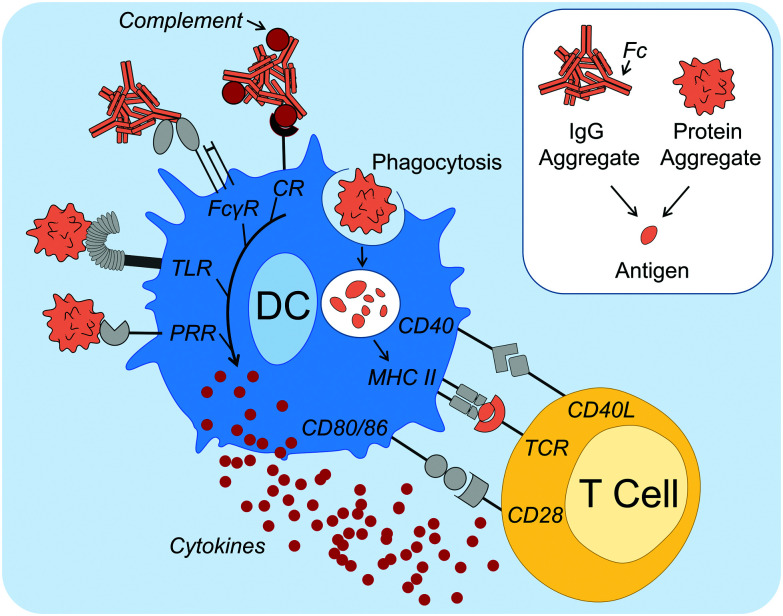
Dendritic cell (DC) activation and maturation mechanisms induced by protein aggregates. DC activation and maturation induced by protein aggregates has been linked to PRR signaling, including TLRs, as well as FcγR and complement activation by antibody aggregates. Association with danger leads to aggregates being taken up, processed and peptides (antigens) presented on MHC II, as part of DC maturation. Mature DCs also secrete cytokines and have upregulated expression of co-stimulatory molecules, such as CD40, CD80 and CD86. Antigen-specific T cells become activated upon recognition of all three signals (antigen, co-stimulatory surface molecules and cytokines) and in turn can aid antigen-specific B cell activation and differentiation into ADA-producing plasma cells. Abbreviations: ADA, anti-drug antibody; CD, cluster of differentiation; CR, complement receptor; FcγR, Fcγ receptor; L, ligand; MHC II, major histocompatibility complex class II; PRR, pattern recognition receptor; TCR, T cell receptor; TLR, Toll-like receptor. Servier Medical Art PowerPoint image bank was used to construct this diagram.

Concerning the mechanisms of how protein aggregates can induce DC maturation and downstream responses (Fig. 1), in the case of IgG antibody therapeutics, the role of the Fc gamma receptors (FcγRs) and complement activation have been implicated. Antibodies are composed of an antigen-binding region (called Fab) and a functional region (called Fc). FcγRs are receptors that bind to the Fc region of IgG antibodies, and are found on many immune cells, including DCs. Cross linking of FcγRs upon binding to Fc augments DC phagocytosis, maturation and antigen presentation.^39^ Moreover, aggregation of therapeutic antibodies has been shown to enhance binding to FcγRs.^32,40^ Stirring induced aggregates of infliximab, rituximab, panitumumab and natalizumab were all shown to enhance binding to various FcγRs as shown by reporter cell lines.^40^ Moreover, Joubert *et al.* demonstrated that three model human IgGs, aggregated by stir-stress, enhanced PBMC secretion of the pro-inflammatory cytokines IL-1β, tumor necrosis factor alpha (TNFα), IL-6, macrophage pro-inflammatory protein (MIP)-1α and MIP-1β.^32^ Moreover, in the former study it was also demonstrated that inhibition of complement opsonization attenuated aggregate-induced cytokine secretion. The complement system can be activated by the Fc regions of IgM and IgG antibodies *via* the classical pathway,^41^ potentially leading to protein aggregates being coated in complement proteins. This is known as complement opsonization: recognition by complement receptors on innate immune cells, such as DCs, leads to phagocytosis of the opsonized particle. It is therefore a likely contributor to immune activation by antibody aggregates.

Regarding DC activation mechanisms that cannot be attributed to the antibody Fc region, Joubert *et al.* further demonstrated potent attenuation of cytokine secretion upon Toll-like receptor (TLR) 4 or TLR2 inhibition.^32^ TLRs are one of several families of PRRs and they recognize conserved molecular patterns derived from microbes.^29^ Other studies that support the hypothesis that PRR signaling can be involved in protein aggregate activation of immune responses have shown that aggregated hGH,^33^ infliximab^34^ and intravenous immunoglobulin (IVIG)^30^ induce signaling components involved in PRR signaling, including nuclear translocation of nuclear factor κB (NFκB).^33^ NFκB is a nuclear transcription factor that is essential for the induction of DC inflammatory responses.^42^

In summary, these studies have identified that DC maturation is a possible mechanism for protein aggregate induced immune activation. DC maturation is critical for the induction of downstream adaptive immune responses against an antigen, including when breaking tolerance towards native proteins (autoantigens).^43,44^ Although a global mechanism for immune activation is unlikely, growing evidence suggests that aggregates can induce one or more danger signal(s), possibly *via* PRR and/or FcγR recognition, as well as complement activation, thus overcoming immune tolerance. Protein aggregates have been shown to activate innate immune responses, however to what extent adaptive immune responses are altered, including the production of ADAs, remains to be addressed.

### Activation of adaptive immunity

To investigate the activation of adaptive immune responses, the most useful experimental models are those that take immune tolerance into account. With regard to murine studies for instance, there is a good argument in favor of the use of transgenic (TG) mice. These are mice that express the human ortholog of the protein of interest and therefore establish central tolerance towards it. Both T cells and B cells go through negative selection processes during development to eliminate autoreactive cells that recognize native epitopes;^45,46^ a process referred to as central tolerance. For T cells, there is also active maintenance of peripheral tolerance in the form of regulatory T cells (Tregs).^46^ TG mice can therefore be used to study which protein formulations are immunogenic enough to overcome these tolerogenic mechanisms. By contrast, work with wild type (WT) animals can give misleading results regarding the immunogenicity of protein formulations. For example, when the immunogenicity of stress-induced aggregation formulations of recombinant human IFN alpha (rhIFNα)2b^47,48^ or rhIFNβ^49,50^ were compared to the monomer, in WT mice it was shown that the monomer was enough to induce ADA production, whereas in the TG mice only formulations with aggregates resulted in ADA induction. As a tolerogenic environment towards the protein in question is clearly crucial to investigate aggregate immunogenicity, this section will focus on both clinical and animal work, where tolerance is established.

As described by Moussa *et al.*, the primary question regarding adaptive immune responses has been whether the induction of ADA is due to a T cell dependent or T cell independent mechanism.^16^ In the previous section it was demonstrated that protein aggregate induced DC maturation could lead to downstream activation of T cells,^32,35,36,51,52^ which intimates that T cell dependent responses are a possible route. With regard to clinical data, a number of studies have made an association between ADA production and T cell involvement. For instance, when PBMCs were taken from 23 MS patients treated with IFNβ, and T cell proliferation was measured following IFNβ incubation, the T cells demonstrating the highest response rate came from patients that had developed ADA.^53^ Furthermore, in two MS patients that were positive for anti-natalizumab antibodies also had natalizumab reactive T cells.^4^

**Fig. 2:**
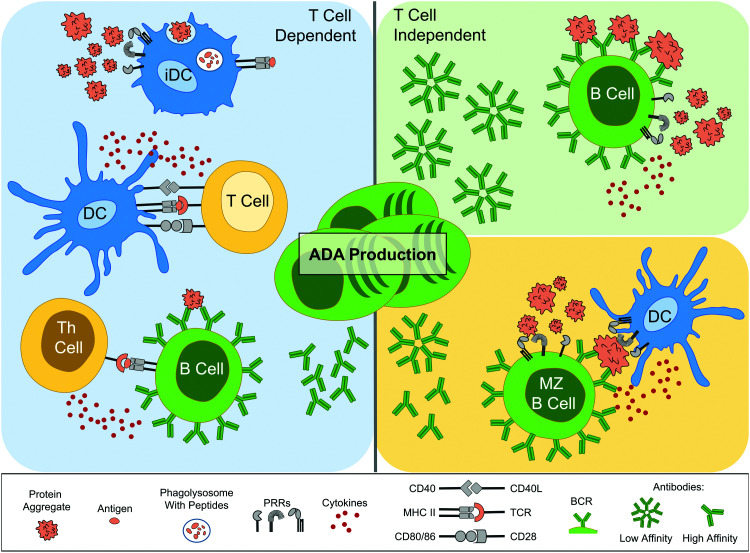
Schematic of the implicated routes leading to anti-drug antibody (ADA) production by protein aggregates. On the left panel, the T cell dependent route is initiated by innate immune cells, such as immature DCs (iDCs), which associate protein aggregates with danger, possibly *via* PRRs. This leads to aggregates being taken up, processed and peptides (antigens) presented on MHC II, as part of DC maturation. Mature DCs also secrete cytokines and have up-regulated expression of co-stimulatory molecules, such as CD40, CD80 and CD86. Antigen-specific T cells become activated upon recognition of all three signals and in turn aid antigen-specific B cell activation and differentiation into ADA-producing plasma cells. Th cells also aid antibody class switching and the development of high-affinity antibodies *via* somatic mutations. The right panels depict T cell independent routes. In the top panel cross-linking of antigen-specific BCRs, along with PRR recognition, leads to activation and differentiation into ADA producing plasma cells, which are restricted to low-affinity IgM antibodies. In the bottom right panel, antigen presentation by innate immune cells, such as DCs, is implicated in activating MZ B cells, which upon PRR recognition, activate and differentiate into ADA producing plasma cells. Abbreviations: BCR, B cell receptor; CD, cluster of differentiation; DC, dendritic cell; MHC II, major histocompatibility complex class II; MZ, marginal zone; PRR, pattern recognition receptor; TCR, T cell receptor; Th, T helper. Servier Medical Art PowerPoint image bank was used to construct this diagram.

*In vivo* experimental results from mice have indicated however that both T cell dependent and independent routes of ADA development can be employed. As an example, low-affinity IgM antibody production is associated with T cell independent B cell activation,^54^ through for instance repetitive antigens causing multiple BCR cross-linking. Murine studies have demonstrated an enhancement of IgM antibody production in response to aggregated proteins compared to their monomer counterpart.^51,52^ Moreover, another investigation where TG mice were injected with Betaferon® – an immunogenic formulation of rhIFNβ-1b, known to have a high degree of aggregation^55,56^ – ADA production was attenuated upon early depletion of marginal zone (MZ) B cells.^50^ DCs can seemingly present antigens to MZ B cells, which are heavily implicated in T cell independent antibody production.^54^ However, these same studies have indicated that T cell dependent responses are also important. For instance, constant depletion of CD4+ T cells, both prior to and after Betaferon® administration, also reduced ADAs in the majority of the mice.^50^ Moreover, protein aggregates have been observed to cause a significant enhancement of murine IgG2a ADA production, an IgG subtype which is dependent upon Th cell induced antibody class switching.^54^ Overall, the combined clinical and *in vivo* work has shown that protein aggregates have been implicated in activating several ADA production routes (Fig. 2).

## Immunogenic aggregation characteristics

In the previous section, the various immune activating pathways shown to be triggered by protein aggregates were discussed. However, it is clear that not all protein aggregates activate immune responses, and moreover the responses induced can vary significantly between proteins. To better understand these discrepancies, this section will discuss how various aggregation characteristics affect immunogenicity.

In the current text, the phrase “protein aggregate” is used to describe a stable complex composed of two or more protein monomers, held together by covalent or non-covalent forces. Aggregates can either form in solution, or on surfaces due to adsorption, and the aggregation process(es) can be either reversible or essentially irreversible. Furthermore, depending on the protein and its environment, aggregates can range from smaller soluble oligomers or multimers, to large insoluble aggregates. Moreover, proteins can either come together to form an unstructured amorphous aggregate, which often has a granular or particulate morphology, however some proteins can self-assemble into highly structured linear amyloid fibrils.^57–59^ This distinctive type of aggregate is important to highlight, as the formation of amyloid fibrils has been identified as a contributing factor in neurodegenerative diseases including Alzheimer's and Parkinson's disease, as well as prion disorders.^60^

Whilst there is no single aggregation pathway, mechanistic studies have highlighted the importance of colloidal and conformational stability.^61,62^ Colloidal stability refers to monomers remaining separated from each other in solution, which depends on factors including solubility, repulsive interactions and on factors governing collision rates, such as concentration. Conformational stability affects whether a protein stays in its folded state, and (partial) unfolding may expose hydrophobic regions which promote protein–protein interactions and thus propagate aggregation.^62–64^

Protein stability and thus aggregation are affected by various stresses that can occur during production, processing, storage and transportation of therapeutic proteins.^57,59^ Such stresses include changes in temperature,^65^ pH and ionic strength^66^ during manufacture and processing, mechanical agitation stresses, such as pumping and shaking during processing and transportation,^67,68^ and freeze-thawing stress from storage.^69,70^ Furthermore, oxidation, which can be caused by UV light exposure^71,72^ or metal contamination,^25,73^ has also been associated with aggregate formation.^74,75^ The stability of the protein itself, as well as the intensity and duration of one or more stresses affects the size, concentration, solubility, charge and morphology of resulting aggregates, as well as the extent of chemical modifications, unfolding and degradation. Given this breadth of possibilities, it is hardly surprising that laboratory research has demonstrated that practically every stress mentioned thus far can catalyze the formation of potentially immunogenic aggregates. Whilst it is therefore extremely difficult to predict whether one stress is more likely to produce immunogenic aggregates over another, this section will consider what physicochemical properties of aggregates may increase their immunogenicity.

### Immunogenicity of individual proteins

Individual protein monomers vary regarding their intrinsic immunogenicity and correspondingly to the resulting/total immunogenicity of their aggregates. For example, when aggregate formulations of infliximab and natalizumab were induced by either shear- or heat stress, only aggregated formulations of infliximab enhanced MoDC CD83 and CD86 expression and secretion of the cytokines IL-6, IL-1β, IL-8, TNFα and IL-12p40.^34^ Furthermore, when two model human IgGs were forcibly aggregated by a combination of heat and shaking stress, both were able to induce MoDC maturation, but one gave markedly greater enhancement of CD80, CD86 and CD83 compared to the other.^31^ Furthermore, the individual protein also affects the type of immune response induced. For example, when stir-induced aggregates of hGH, rituximab and a serum-purified human IgG were investigated for their elicited immune responses, their induced profiles were somewhat distinct.^35^ While all aggregate formulations enhanced MoDC secretion of IL-8, CXCL10, CCL4 and IL-12p40, only aggregated hGH also increased the secretion of the chemokines CCL2 and CCL3. That the type of immune response elicited varies depending on the protein involved is further supported by a study where samples of rituximab and trastuzumab were aggregated by a variety of stresses: stirring, heating or freeze-thawing.^36^ Here, all of the aggregates of rituximab, regardless of the stress that induced them, enhanced PBMC secretion of TNFα whereas the trastuzumab aggregates all enhanced the secretion of IL-2 and IL-10 by PBMCs, highlighting the discrete difference in immune response type to the two different aggregates.^36^ Given the diverse range of stresses involved in inducing the aggregate formulations, it is likely that the differing total immunogenicity of the protein aggregate formulations were caused by the immunogenic properties of the protein monomers that comprised the aggregates.

Apart from inherent/intrinsic immunogenicity differences, another factor that differs between individual proteins is their propensity to aggregate, and how they aggregate. Several factors contribute to these differences, including differences in conformational stability – some proteins are more prone towards unfolding than others – or colloidal stability, where the degree of repulsive forces can vary.^61^ Differences in protein propensity to aggregate was demonstrated practically in a study where two monoclonal human antibodies, of 148 kDa and 204 kDa in size respectively, were subjected to thermal stress: only the former of these antibodies aggregated under this type of stress, whereas both aggregated in response to shaking stress.^51^ These differences in propensity to aggregate can also affect the overall composition of the resulting aggregate formulation. For example, when three therapeutic antibody formulations (rituximab, panitumumab and natalizumab) were stressed by stirring for 20 hours apiece, the composition of the resulting formulations differed markedly.^40^ Whereas both the stressed formulations of rituximab and natalizumab were mostly composed of aggregates in the 5–12 μm range, with the majority at the upper end, the formulation produced by stressing panitumumab showed greater polydispersity, with a broader range of 0.5–10 μm-sized aggregates, and the majority contrastingly in the lower 1–2 μm range.^40^ In summary, the protein itself affects the intensity and type of immune response that the resulting aggregates can elicit, as well as its stability in solution, which is a determining factor for aggregation initiation and composition.

### Conserved conformation

Adaptive immune responses, including the production of ADAs and especially neutralizing antibodies, target specific epitopes/antigens. However, conformational stability of the protein and various stresses can exacerbate protein unfolding together with aggregation.^57^ A high degree of unfolding yields “non-native” aggregates that have a distinct 3D structure compared to native monomer fold. Furthermore, stresses can also cause chemical modifications which further alter the native protein structure. Since severe structural changes can occur during aggregation processes, it is expected such changes affect the aggregate formulations immunogenicity, as well as the subsequent immune responses targeted towards the native protein therapeutic.

That changes in structure potentially alters the range of antigens targeted by the immune response has been demonstrated by considering which antigens are presented by DCs. It has for instance been demonstrated that protein aggregation can increase the number of epitopes presented by MHC II.^31^ When an MHC associated peptide proteomics (MAPPs) assay was carried out, comparing aggregated and non-aggregated formulations of two human IgGs, it was found that heat and shake induced aggregation increased the number of presented epitopes on MoDCs 16- and 5-fold for the two proteins compared to each respective non-aggregated formulation.^31^ This suggests that structural changes induced by aggregation may lead to immune responses targeting antigens either not present (due to chemical modification) or unavailable on the therapeutic protein (hidden by native fold). This hypothesis is further supported by work using humanized single chain variable antibody fragment (scFv), whereby the use of peptide mapping and ELISA, a peptide sequence was identified to be highly targeted by aggregate induced antibodies, but not monomer induced antibodies in WT mice.^63^ Using protein structure prediction software (I-TASSER), molecular dynamics simulations and simulated annealing, it was determined that this peptide was found in a hydrophobic domain of scFv, most likely becoming exposed by partial unfolding. Furthermore, Fathallah *et al.* demonstrated that while native aggregates were found to enhance ADAs, the non-native aggregates induced ADA levels similar or lower than those induced by the monomer, suggesting that native conformation is important for immunogenicity against the native protein.

Chemical modification has also been associated with altered immunogenicity of protein aggregates. For instance when rhIFNα2a was stressed *via* glutaraldehyde cross-linking, this process reduced the immunogenicity of resulting protein aggregates.^48^ However, in stark contrast, a study by Boll *et al.* demonstrated that chemical modification *via* oxidation can enhance immunogenicity.^71^ Aggregate formulations of a human IgG were made employing three types of oxidation stress and ADA production against the native protein was investigated in TG mice. A correlation was found between enhanced ADA production and higher degree of chemical modification (as measured by liquid chromatography and mass spectrometry [LC-MS] or capillary sodium dodecyl sulfate electrophoresis [CE-SDS]).^71^ Relating this back to the hypothesis that protein aggregates may interact with one or more PRRs, it may be the case that certain chemical modifications could enhance affinity for such interactions. Taken together, these studies suggest that overall, a certain degree of structural conformity is required to elicit immune responses against a native protein, however some degree of (chemical) modification may not be a hindrance to such immune responses, and may even enhance them.

### Aggregate size and particle number

Aggregation size is a property that can affect a number of factors, such as distribution,^9^ uptake^36^ and bio-available surface area. Whilst aggregation itself is associated with enhanced uptake, by for instance MoDCs,^36^ the aggregate size may impact both the extent and route of uptake. Studies with particles have shown that larger particles (≥1 μm) are taken up by micropinocytosis, whereas smaller particles are taken up by endocytosis.^76^ Route of uptake could affect aggregate intracellular degradation, processing, as well as antigen presentation. With regard to the extent of uptake, some studies have highlighted that smaller particles are taken up to a greater extent than larger particles. For example, when inert polystyrene particles (for which size and polydispersity is more easily controlled than it is for protein aggregates), of either 50 nm or 500 nm in size, were administered to naïve mice intratracheally, the 50 nm particles were preferentially taken up by lung innate immune cells, including DCs.^77^ A similar comparison between 20 nm and 1 μm polystyrene particles showed that the 20 nm particles were taken up to a greater extent by bone marrow derived dendritic cells (BMDCs).^78^ In the former example, particle exposure of either size enhanced bronchoalveolar lavage (BAL) fluid levels of the cytokines IL-6 and IL-12p40 – cytokines that have also been shown to be induced by protein aggregates^34,35^ – however the smaller (50 nm) particles induced significantly higher levels than the larger particles.^77^

Regarding work with proteins, studies have identified a broad immunogenic range of aggregate sizes of roughly 50 nm–5 μm. Because aggregate formulations are commonly composed of particulates of a broad range of sizes, even if the average particle size is reported, the influence of smaller or larger particles are difficult to account for. As such, very few studies using aggregates can be used to accurately study the influence of particle size, even when comprehensive characterization is carried out. However, for the majority of reported studies, aggregate size is intrinsically linked with particle number, and overall there appears to be a correlation between higher particle number and greater immunogenicity. For example, murine studies comparing ∼0.2 μm and ≥1 μm aggregates of a model murine antibody^79^ and 0.5–2 μm and ∼2–8 μm aggregates of rhIFNβ-1b,^80^ both found that the more immunogenic formulations (as measured by ADAs), were comprised of smaller aggregates, where the particle count was roughly four and five times higher respectively (as measured by nanoparticle tracking analysis [NTA] and microflow imaging [MFI]). The relevance of particle number is also supported by another study, a model human IgG was subjected to various stresses: stirring (20 hours and 3 days), shear stress as well as high temperature with basic pH. With these aggregate formulations they identified that increased particle number in the 2–10 μm size range correlated positively with enhanced PBMC cytokine secretion.^32^

Overall, the work carried out thus far has identified a broad range of immunogenic aggregate sizes that can overcome tolerance. Importantly, because the majority of reported data compare formulations with the same mass of protein, aggregate size as a factor cannot be separated from particle count or total surface area. Cumulatively, it can be concluded that smaller aggregates with high particle number (and surface area) tend to be more immunogenic than larger aggregate formulations. For future experimentation, to account for each of these possibly immunogenic factors, other experimental approaches would have to be employed, approaches that account for total surface area and/or particle number, or alternatively approaches that saturate the immune responses induced by each size of aggregate to see if one has a greater maximum induction than the other. With these experimental designs it would be possible to investigate whether certain size ranges are inherently more immunogenic than others, and the immune mechanisms involved.

### Aggregate morphology

Another aggregate characteristic that can affect surface area is morphology. Aggregate morphology can be influenced by the protein, but is also thought to be affected by the type of stress afflicted.^57^ Although this is a characteristic that is often overlooked, some evidence can be linked to morphology affecting total aggregate immunogenicity. For instance when the immunogenicity of globular and filamentous aggregates, of two model human IgGs, were compared *via* MoDC maturation, the filamentous aggregates induced the highest expression of CD86, CD80 and CD83.^31^ The different morphologies were induced by different stresses; where a combination of heat and shaking induced the filamentous aggregates, whereas freeze-thawing or shear stresses induced more globular aggregates (as seen *via* MFI). A globular shape has a much lower surface area compared to its mass/volume than an elongated shape, therefore, this observation supports the hypothesis that enhanced surface area may enhance immune activation. While more research is required to support this hypothesis, the correlations between smaller aggregate size and particle concentration (with accompanying greater surface area) with enhanced immune activation would indicate that it is a possibility.

**Fig. 3:**
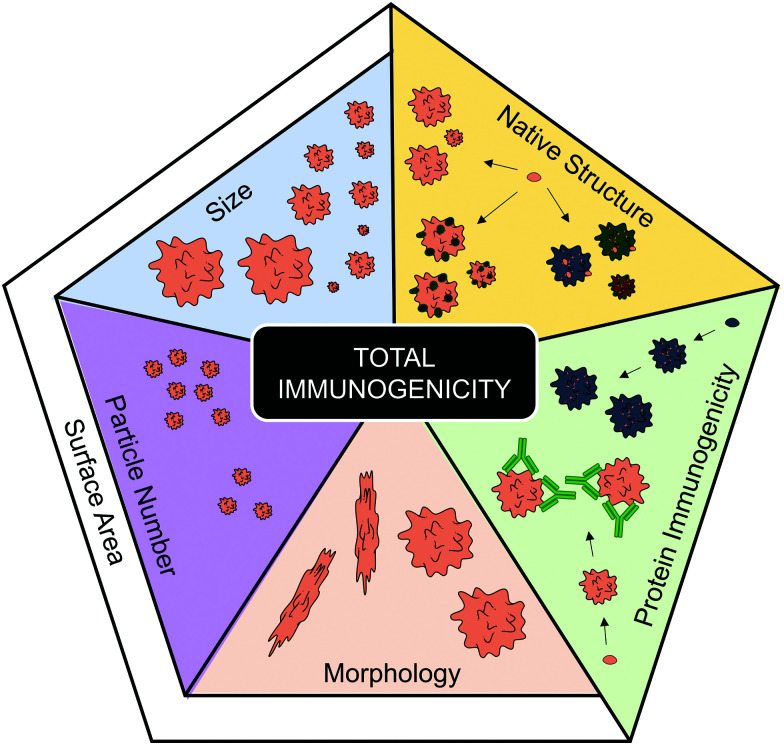
Characteristics that have been implicated in contributing to total aggregate immunogenicity. The protein immunogenicity fraction highlights how individual proteins (represented by different colors) and their corresponding aggregates can vary regarding their intrinsic immunogenicity. The native structure fraction represents that the degree of structural modifications (represented by darker colors), such as unfolding or oxidation, occurring during aggregation can affect the resulting total immunogenicity of the aggregate formulation. Regarding the fractions pertaining to physical aggregation properties; size, particle number and morphology have all been implicated to contribute to overall aggregate immunogenicity. The research regarding these three latter factors indicate that a high total surface area increases total aggregate immunogenicity. Servier Medical Art PowerPoint image bank was used to construct this diagram.

Although the role of protein aggregation in causing disease falls outside the scope of this review, it is nevertheless worthy of note that the formation of linear amyloid fibrils is a key driver of Alzheimer's (β-amyloid)^81^ and Parkinson's disease (α-synuclein).^82^ These are both neurodegenerative diseases where unwanted immune activation is a key component of the pathology.^83,84^ However, it should be noted that studies have identified that neurotoxicity associated with the formation of these fibrils is caused by accumulation of prefibrillar intermediates, rather than mature amyloid fibrils.^85–87^ In summary, this section has identified a number of aggregate characteristics (Fig. 3) that potentially contribute to enhanced immunogenicity.

## Strategies for prevention of protein aggregation

Having considered the immune activating properties of protein aggregates and some of the physicochemical characteristics that affect them, this section will consider mechanisms that induce and propagate aggregation in more detail, as well as strategies used to minimize or avoid aggregation. As discussed earlier in the review, conformational and colloidal stability have been identified as key determinants of protein aggregation.^61,62,88^ How stable a protein is in both regards depends on the protein in question and on its environmental conditions. To control protein stability, both during production and in the final formulation of the therapeutic, environmental factors such as pH, ionic strength, temperature and mechanical agitation need to be considered, as well as the addition of excipients in the formulation.^89^ Excipients are all the components of a drug formulation other than the active drug itself, including salts and buffer system to control pH and osmolality, as well as other added compounds that for instance reduce viscosity,^90^ act as preservatives^88^ or that enhance protein stability through a variety of mechanisms.^91,92^

### Controlling sources of stress

Environmental conditions during production, processing, transportation and storage of a therapeutic protein can enhance aggregation by affecting either (or both) conformational and colloidal stability. With regard to conformational stability, temperature can play a major role. Denaturation occurs more frequently when the temperature approaches either the melting/transition temperature (*T*_m_) or the cold denaturation temperature – at either point half is in an unfolded state.^70,93,94^ Conformational stability has also been shown to be affected by other stresses, including changes in pH^94^ and ionic strength.^95^ Furthermore, changes in conformation caused by adsorption onto surfaces, such as ice crystals during freezing,^96^ or air bubbles formed by mechanical agitation,^57^ is proposed to lead to protein denaturation.^97,98^

With regard to colloidal stability, factors that crucially affect repulsive interactions between protein monomers include ionic strength and pH. When colloidal stability *via* electrostatic repulsion is a key determinant of aggregation, increased ionic strength has a de-stabilizing effect,^74,93,99^ due to charge-screening and the consequent reduction of the Debye length that characterizes the span of electrostatic repulsion. pH affects the overall charge of the protein by regulating the density of protonated groups, thereby also affecting intra and inter-molecular electrostatic repulsions.^93,100^

To prevent aggregation, these environmental factors therefore need to be considered during manufacture and formulation development. As an example, protein therapeutics are often lyophilized to keep them stable during storage and transportation, a process that can result in aggregation *via* cold denaturation^69,70^ or by surface interaction with the ice–water interface.^96^ However, the lyophilization process and/or formulation can be optimized, to minimize aggregate formation. To prevent aggregation during freeze-drying a recent study demonstrated how changing to a fast freezing procedure reduced aggregation and loss of activity of a model protein (myoglobin), which is sensitive to cold denaturation.^96^ This was hypothesized to be due to fast-freezing reducing the time in a cold solution, where cold denaturation occurs. However, this quick procedure causes ice to form as small ice crystals, leading to a large surface area for ice–water interactions, and would therefore be less ideal for proteins susceptible to conformational instability at surfaces. Indeed, with the use of model protein lactate dehydrogenase, it was demonstrated that the fast-freezing procedure, which was effective for myoglobin, was detrimental for lactate dehydrogenase stability, leading to aggregation and loss of activity.^96^ This study effectively demonstrated how protein stabilities can vary to a great extent, and adjusting their environmental parameters accordingly can prevent potentially immunogenic aggregation formation.

In addition to the rate of freezing, another factor that can affect stability for lyophilized proteins is the addition of an annealing step,^101^ where (post-freezing) the formulation is warmed to a subfreezing temperature, above the gas transition temperature for the formulation, and held there for a time before the temperature is dropped once again.^102,103^ Annealing has been shown to increase ice crystal size and enhance subsequent drying rate and efficiency.^103,104^ Inclusion of an annealing step has further been shown to better retain the native protein fold,^105^ reduce aggregation during storage^105,106^ and reduce the formation of bubbles upon re-suspension.^105^ However, optimal annealing conditions need to be identified for each protein, and in fact annealing may not be beneficial in all cases, as it has also been reported that annealing can contrastingly augment aggregation.^107^ Overall, environmental conditions significantly contribute to protein stability and thus carefully investigating such conditions for each protein formulation reduces the risk of the formation of immunogenic aggregates.

### Influence of additives on protein stability and aggregation

#### Sugars: sucrose and trehalose as examples

Apart from controlling environmental sources of protein stress, there are numerous excipients that hinder protein denaturation, for instance disaccharides, such as sucrose^62^ and trehalose.^90^ In solution these are osmolytes and act as stabilizers by preferential exclusion;^108–110^*i.e.* by creating a highly polar environment surrounding proteins, thus inhibiting the exposure of hydrophobic pockets hidden by the native fold. This shifts the conformational equilibrium towards a more compact species, thus making unfolded states less thermodynamically favourable.^108,109,111^ This enhanced stability can be measured by an increase in protein Tm. Studies on model proteins demonstrated that the addition of trehalose^110^ or sucrose^109^ increased the Tm of each protein in a concentration-dependent manner. Most importantly, the addition of these conformational stabilizers has been shown to reduce protein aggregation in solution.^62,112^

These two disaccharides moreover act as stabilizers during freezing and lyophilization, although *via* somewhat distinct mechanisms. During lyophilization of hGH, sucrose has been shown to better promote conformational stability *via* preferential exclusion, which prevents interaction with ice–water interfaces, leading to reduced aggregation during dry-freezing.^113–115^ However once dry, trehalose has been shown to better preserve native conformation and prevent protein–protein interactions^115^ by forming a glass matrix:^116,117^ a rigid matrix that immobilizes protein monomers, thus preventing their denaturation and movement, and thereby also their aggregation.^92,118,119^ However, one potential issue with the use of lyo-/cryo-protectants is that they can become separated from the protein upon freezing. A study working with formulations of albumin and trehalose, found that albumin accumulated at the ice interface, thus physically separating from the trehalose.^119^ To avoid this issue, it was found that there was a range of freezing temperatures where separation did not occur, and that the range narrowed with increasing trehalose concentration.^119^ Overall, these studies have demonstrated that excipients, with the disaccharides sucrose and trehalose as examples, can act as stabilizers.

#### Preservative: benzyl alcohol

Benzyl alcohol is one of the most common antimicrobial preservatives used in multidose protein formulations,^92^ however, it has been demonstrated to promote unfolding and aggregation of for instance recombinant human IL-1 receptor antagonist (rhIL-1RA),^88^ recombinant human granulocyte colony-stimulating factor (rhGM-CSF),^112^ hGH^120^ and IFNγ.^121,122^ Why this aggregation occurs was investigated with rhIL-1RA, where it was shown that benzyl alcohol preferentially binds to an aggregate prone, partially unfolded species *via* hydrophobic interactions, thus reducing conformational stability, as shown by reduced Tm^123^ and overall driving aggregation.^123,124^ Benzyl alcohol is therefore an example of how certain excipients can reduce protein stability, a factor that must be taken into consideration during development of biotherapeutics.

#### Surfactants

Surfactants are another type of excipient than can protect against different types of stress. Non-ionic surfactants are often used in protein formulations to prevent aggregation caused by adsorption onto surfaces and are thought to work by out-competing the protein for access to hydrophobic interfaces.^92^ Such surfaces include interaction with the vessel holding the formulation, but also adsorption to ice–water interfaces during freezing^96^ and to air–water interfaces introduced by for instance mechanical agitation stresses.^125^

The most common non-ionic surfactants used in protein formulations are polysorbate 20 (PS20) and polysorbate 80 (PS80).^92^ PS20 and PS80 are composed of two parts: a hydrophilic head of poly-(oxyethylene) (POE) and dehydrated sugar (sorbital) esters and various hydrophobic fatty acid tails.^126,127^ Due to their amphipathic nature, they resolve hydrophobic surfaces^105^ thereby blocking the binding, denaturation and aggregation of proteins at these surfaces.^128^ This maintenance of conformational stability by either PS20 or PS80 has been shown to prevent aggregation caused by mechanical agitation^67,128–130^ and during freezing.^96,131^ However, commercial polysorbate preparations are often prone to spontaneous autooxidation, resulting in the formation of peroxides, which can damage proteins (see section on Chemical degradation).

### Influence of structural modifications on protein stability and aggregation

#### Chemical degradation

Another aspect of stresses that induce and/or propagate aggregation is that they can simultaneously cause covalent modifications, such as oxidation, deamidation, disulfide bridge formation and cross linking.^62,132,133^ Structural changes induced by chemical modification can alter the native fold of proteins, as well as change intermolecular interactions, which may affect aggregation, immunogenicity and impede the function of therapeutics.^75,134^ For example, deamidation is a hydrolysis reaction that affects asparagine and glutamine residues, which can be catalyzed by heat^133^ or changes in pH.^135,136^ The hydrolysis results in the formation of aspartic acid, isoaspartic acid or glutamic acid, and correspondingly the loss of a positively charged functional group. This may not only affect colloidal stability, and thereby aggregation, but could also affect interactions with immune cells.

Of the potential chemical modifications to protein structure, one the most common is oxidation,^134^ which – depending on the site of oxidation and the location of the residues – can promote both aggregation and enhance immunogenicity. Amino acids that are susceptible to oxidation include methionine and tryptophan, cysteine, histidine, tyrosine and phenylalanine.^137,138^ Randomized oxidation, has in turn been associated with reduced conformational stability and increased aggregation.^74,75^ More importantly, oxidation has also been demonstrated to enhance the immunogenicity of protein aggregates.^71^ A range of stresses are known to cause oxidation along with immunogenic aggregation, such as UV light exposure,^72^ metal contaminants,^133,139^ heat^132,139^ and even mechanical agitation.^35,133^ Moreover, excipients can cause oxidation, for instance polyoxyethylene-based surfactants (PS20 and PS80) contain ether linkages and unsaturated alkyl chains that can auto-oxidize to form reactive alkyl peroxides, which in turn cause oxidative changes to the protein.^140,141^ To prevent oxidative protein changes, the use of chelators^92,142,143^ to prevent metal-induced oxidation or antioxidants^40,90,137,144,145^ have been demonstrated to impede protein oxidation and aggregation.

#### Antibody drug conjugates (ADCs)

**Fig. 4:**
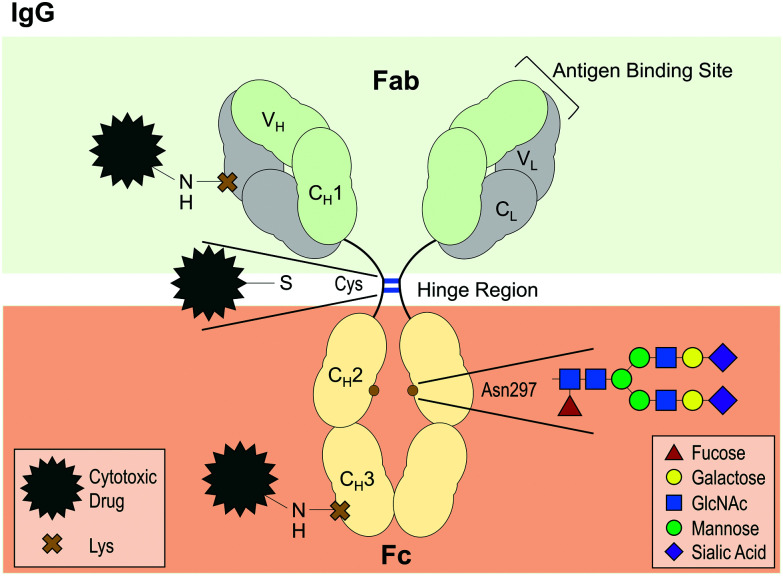
Diagram of IgG antibody structure, highlighting the highly conserved glycosylation site at asparagine (Asp) 297 and examples of conjugation sites for the design of antibody drug conjugates (ADCs). IgG antibodies are composed of two light chains (L) and two heavy chains (H), each with a variable domain (V) for antigen binding and one (L) or three (H) constant domains (C). The antigen-binding fragment (Fab) is linked to the crystallisable fragment (Fc) *via* a hinge region, where the two heavy chains are linked together by two disulfide bonds (blue lines). For ADC conjugation, disulfide bonds, such as those in the hinge region, can be reduced to allow attachment of a cytotoxic drug. Another conventional method is to use for instance a reactive ester modified form of the drug to conjugate it to random lysine (Lys) residues, which are present in every domain. Servier Medical Art PowerPoint image bank was used to construct this diagram.

Protein stability can also be affected by intentional structural modifications. For example, antibody drug conjugates (ADCs; Fig. 4) are a class of antibody-based therapeutics – where cytotoxic small molecules/drugs are conjugated onto antibodies to target cancers^146^ – that have been demonstrated to be more prone towards aggregation than their non-conjugated counterparts.^146–151^ Conjugation of either auristatin^147,148^ or emtansine^146,149^ to IgG antibodies *via* cysteine or lysine residues respectively, has been shown to enhance aggregation upon heat stress. Furthermore, it has been demonstrated that this destabilizing effect is heavily dependent upon drug to antibody ratio, where instability was correlated with a higher drug ratio.^148,150,151^

Regarding why conjugation augments protein destabilization and aggregation, a number of factors have been identified. By investigating transition temperatures of different antibody regions, it was identified that the CH_2_ domain (on Fc) was particularly susceptible to conformational instability following conjugation with either cytotoxic molecule.^148–150^ Moreover, the conjugation of auristatin has been shown to enhance the hydrophobicity of the resulting ADC compared to its non-conjugated counterpart, thus directly reducing colloidal stability.^147^ Furthermore, when attaching emtansine to the amino group of lysine, forming an amide bond, such conjugation results in the loss of positive charges and can thus negatively affect colloidal stability.^146^ Trastuzumab conjugated with emtansine was found to have a lower net charge and reduced electrostatic repulsion than its non-conjugated counterpart.^146^ This was thus proposed as a key determinant as to why the ADC aggregated to a greater extent than trastuzumab when exposed to heat stress.^146^ Taken together, ADCs are examples of how intentional chemical modifications can significantly alter a protein's physicochemical properties, stability and its propensity to aggregate.

#### PEGylation to enhance stability

A common modification of biotherapeutics is the conjugation of polyethylene glycol (PEG), known as PEGylation. Clinical examples of PEGylated biotherapeutics include antibodies, hGH and IFNα.^152^ This is carried out primarily because PEGylation enhances the half-life of a protein therapeutic in plasma,^153–155^ thus enhancing their efficacy. As an example, PEGylation of IFNγ increased the half-life 20-fold and was significantly more effective than non-PEGylated IFNγ at preventing growth of a human tumor xenograft in athymic (no T cells) mice.^153^ This reduction in clearance rate is considered to arise due to the PEG preventing protease-mediated degradation. Furthermore, the increased size of the PEG conjugate impedes glomerular filtration and subsequent excretion in the kidneys.^156^

PEGylation has also been shown to reduce aggregation induced by changes in pH and heat^157^ or induced by the preservative benzyl alcohol.^158^ How PEGylation affects protein stability is not fully understood.^156^ However, simulations with insulin showed that conjugated PEG interacts with the protein surface through hydrophobic interactions, and formed hydrogen bonds with the surrounding water, which was proposed to enhance conformational stability, as shown by increased *T*_m_.^159^ Furthermore, for the model protein adnectin and a domain antibody (dAb), PEGylation has been demonstrated to enhance thermal stability.^157^ However, in the case of chymotrypsinogen, whereas PEGylation protected from benzyl alcohol induced aggregation, it did not enhance thermal stability, demonstrating its effects can vary depending on the protein's identity.^158^ The stabilizing effects of PEGylation have moreover been shown to depend on the length of the PEG chain,^155,158,160^ and the location of the conjugation site.^154,155,161^

Perhaps due to the observed protection against aggregation, PEGylation has also been shown to reduce immunogenicity of proteins. For instance, PEGylation of either IFNβ^154^ or hGH^155^ reduced induction of ADA in rats as compared with their non-PEGylated counterparts. However, despite early assumptions that PEG was indeed non-immunogenic or poorly immunogenic,^152,162^ it has become apparent that immune responses against PEG (including anti-PEG antibodies) is a growing issue.^152,163^ What is especially troubling is that anti-PEG antibodies have not only been detected in patients receiving a PEGylated drug, but also in naïve patients.^152,164^ This growth of anti-PEG immune responses is thought to stem from enhanced exposure to PEGylated products used in a broad variety of applications, including cosmetics and processed foods. As such, the growing frequency of anti-PEG immune responses, and concerns of how these may impact therapeutic efficacy and induce adverse effects, has resulted in a need to find safer options for future drug development.^165–167^ A possible alternative and more biocompatible option to PEGylation is glycosylation.

#### Glycosylation on stability and function

Proteins undergo multiple co- and post-translational modifications which affect both their function and stability; one of the most common modifications is glycosylation.^168^ Glycosylation of proteins can either be *N*- or *O*-glycosylation, where the sugar is connected *via* either a nitrogen or an oxygen atom of an amino acid respectively. Both colloidal^169^ and conformational^170,171^ stability has been shown to be enhanced by glycosylation, which results in reduced aggregation.

With regard to IgG antibodies, there is a highly conserved *N*-glycosylation site on CH_2_ domain of the Fc region (Fig. 4).^172^ Glycosylation at this site has been linked with enhanced antibody–receptor interactions, protection against protease degradation and reduced propensity to aggregate.^168,170,173^ These effects appear to depend on the composition and size of the glycan.^168^ For instance, partial de-glycosylation from a branched structure composed of 10 or more monosaccharides to either a monosaccharide or a disaccharide moiety was shown to increase unfolding and aggregation upon heat stress.^168^ Moreover, complete de-glycosylation has been demonstrated to abolish FcγR binding, reduce thermal stability of the CH_2_ domain^170^ and enhance aggregation upon heat stress.^173,174^ Similarly, glycosylation has been shown to protect against acid-induced CH_2_ domain unfolding and subsequent aggregation.^175^ Given the importance of glycosylation for antibody function and stability, recombinant antibodies are usually produced in mammalian expression platforms, such as Chinese hamster ovary (CHO) cells or murine NS0 cells to help retain the native glycosylation pattern as much as possible.^168,176^ Loss of glycosylation in CHO cells has been reported to lead to antibody aggregate accumulation during the bioreactor culture,^177^ demonstrating once more the role of glycosylation for preventing aggregation.

Due to the link between glycosylation and enhanced protein stability, using it as a strategy to prevent aggregation has been widely investigated. Chemical glycosylation of insulin has been demonstrated to impede protein self-association whilst retaining *in vivo* biological activity.^169^ Similarly, chemical glycosylation of model protein α-chymotrypsin with a branched glucan dextran protected against heat-stress induced aggregation.^178^ Protection from aggregation was augmented further with increased glycosylation overall, whereas the use of a small sugar, the disaccharide lactose, by contrast was unable to convey similar stabilizing properties, highlighting that both the size of the glycan and the extent of glycosylation are factors that affect stability.^178^ Chemical glycosylation however, while useful for accessing homogenous glycoproteins on a small scale, is not yet practical for large scale manufacturing or production. Apart from chemical glycosylation, another approach is to design mutein variants of the protein of interest, introducing new glycosylation sites. This was performed for instance with the therapeutic antibodies adalimumab^179^ and bevacizumab^180^ where various *N*-glycosylation sites were introduced in the Fab region of the antibodies. Some of the resulting muteins were shown to enhance the Tm of the Fab region and impede aggregation induced by heat stress.^179,180^ Moreover, hyperglycosylation did not impede Fc–FcγR interactions for adalimumab^179^ nor bevacizumab Fab binding to its target (vascular endothelial growth factor-a [VEGFa]).^180^ Similarly, introducing four *N*-glycosylation sites to rhIFNα has been shown to protect from aggregation induced by either heat stress or freeze–thaw cycles.^181^

As with PEGylation, glycosylation has thus been demonstrated as a viable strategy to enhance protein stability and prevent aggregation. Moreover, glycosylation has also been demonstrated to improve protein half-life in serum and thereby improve therapeutic efficacy.^182–185^ For instance, *in vivo* studies in rats have shown that the use of a glycosylated rhIFNα mutein reduced the serum clearance rate 20-fold^182^ and a glycosylated mutein of human follicle stimulating hormone similarly extended serum half-life,^184^ compared to their respective non-glycosylated counterparts. The glycosylated mutein of human follicle stimulating hormone furthermore showed significantly enhanced potency *in vivo*, as seen by greater ability to augment the ovarian weight and stimulate the serum estradiol levels.^184^ This increase in half-life results from firstly the reduced propensity to aggregate, given that aggregates are cleared from serum more rapidly than monomers, secondly that glycosylation has been shown to protect against protease degradation^173,186^ and lastly that the increase in molecular weight impedes renal clearance.^182,184^ Overall, although any structural modification needs to be investigated on a case-by-case basis to ensure for instance that biological activity remains intact, these studies nevertheless demonstrate how glyco-engineering is a possible means to enhance protein stability, prevent aggregation and possibly therefore also reduce harmful immunogenicity, whilst also enhancing serum half-life.

Whilst the use of glycosylation has a lot of benefits it is important to note that there are some associated challenges with this approach. Although the majority of studies have shown that glycosylation enhances protein stability and thereby prevents aggregation, there are cases where glycosylation has been shown to have a de-stabilizing effect.^187–189^ As an example, a study investigating glycosylation of a IL-2 mutein showed that glycosylation enhanced degradation rate at acidic pH 4 compared with the un-glycosylated variant.^189^

Another potential complication, which was discussed in a recent review,^176^ can arise from the use of non-human mammalian cells for glycosylation of protein therapeutics: the epitopes *N*-glycolylneuraminic acid and Galα1–3Galβ1–(3)4GlcNAc (αGal).^176^ These are sugar epitopes that most mammals express, but humans do not. Therefore, the use of non-human mammalian cell lines for therapeutic protein production can lead to glycosylation with either of these epitopes. Presumably from exposure *via* animal-sourced foods, humans have circulating antibodies against both motifs,^190–193^ which could subsequently target the glycosylated biotherapeutics. Although very little research has been carried out that explores this possibility, it is nevertheless a concern for future protein therapeutic development.

## Discussion and conclusions

At its core, the use of native proteins has led to fundamentally improved treatment of many severe diseases, prominent examples including MS and cancer. The use of native and native-like proteins is a clever way of harnessing immune tolerance towards self to our advantage to avoid harmful immune activation against therapeutics. However, the propensity of proteins to aggregate has emerged as a serious issue which affects a biotherapeutics’ function and immunogenicity. Immune activation can result in the induction of adverse immune-mediated effects, one of the most problematic being the production of ADAs. As a result, testing for ADAs during clinical trials for new therapeutics has become the standard.^194,195^ Moving forward what is needed is a screening procedure to test for immunogenic aggregates, both during development of new therapeutics and during batch testing. With regard to immune activation mechanisms that lead to ADA production however, both T-cell dependent and independent pathways have been implicated to be induced by protein aggregates. As a result, it is difficult to develop assays to screen for immunogenicity that would be applicable for a broad range of proteins. An assay that has been proposed, is to use DC maturation.^34^ Although DC activation is not directly implicated in all routes of ADA production, their maturation occurs in response to a danger signal, which is an important step for overcoming immune tolerance. Thus, this strategy could prove useful and is worthy of further investigation.

With regard to identifying immunogenic protein aggregates there are numerous factors to consider, such as the immunogenicity of the protein itself. Some degree of conservation of the native protein fold is required for effective ADA production against the drug, and immune activation by aggregates is moreover affected by the conservation of immunogenic epitopes.^32^ Similarly, there is also a precedent for identifying immunodominant epitopes to reduce total aggregate immunogenicity. For instance, analysis of MS patient serum for T cell and ADA responses against rhIFNβ found two immunodominant regions.^53^ In another study, adaptive responses towards natalizumab from two patients were investigated, where it was found, by the use of natalizumab variants, that the ADA bound to same region of natalizumab.^4^ A way to prevent adverse protein and aggregate induced immune activation is therefore to modify such immunodominant regions. In the latter study, a “de-immunized” engineered variant of natalizumab was produced, which lacked the immunodominant peptide sequence, which resulted in no cross-reactivity from natalizumab-recognizing T cells.^4^

Apart from considering the intrinsic immunogenicity of the protein itself, another approach is to investigate which physicochemical characteristics contribute to aggregate immunogenicity, such as size (with particle number) and morphology, which affect the bioavailable surface area. Based on reported data, proteins that are prone to aggregate in a manner that forms many small aggregates, as opposed to few large ones, have been identified as more likely to induce adverse immune activation. Furthermore, although more research is required to confirm whether a fibrillary morphology is more immunogenic than globular; if this is the case, then avoiding protein variants or batches that aggregate with this elongated morphology may be beneficial for avoiding harmful immune activation.

**Fig. 5:**
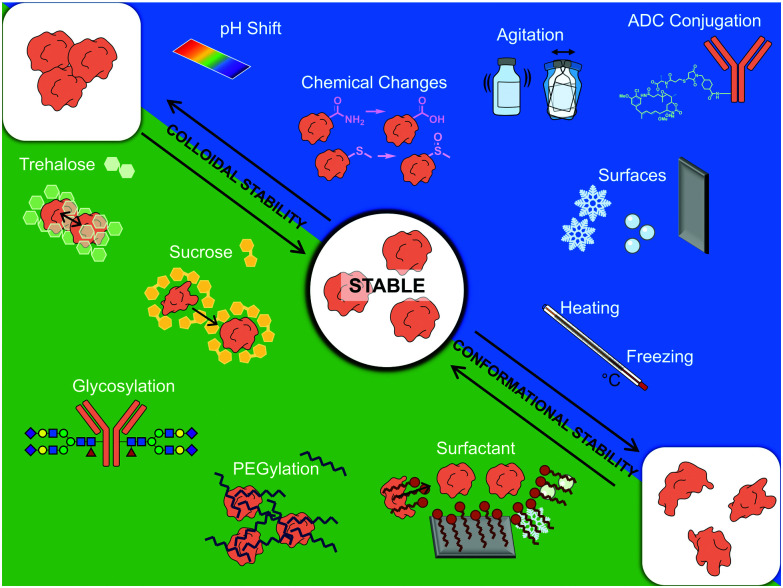
Schematic summary of factors affecting therapeutic protein stability. Servier Medical art PowerPoint image bank was used to construct this diagram.

Along with identifying immunogenic aggregates, numerous strategies have been developed to prevent protein instability during production and distribution of biotherapeutics. These include controlling various stresses during the manufacture, processing, transport and storage, as well as the careful choice of excipients (Fig. 5). Stabilizing excipients include for instance sugars, such as sucrose and trehalose, and surfactants, such as PS20 and PS80. More direct measures to ensure stability include PEGylation and glycosylation. However, with regard to PEGylation and glycosylation there are concerns moving forward regarding the background level of antibody production against both PEG chains and certain glycan motifs. The validity of this concern can be shown with a scenario regarding allergic responses towards cetuximab.^196^ In a study where 25 patients showed hypersensitivity towards cetuximab, it was found that 17 of them produced ADAs prior to treatment. These patients were also shown to have allergies against non-primate mammal allergens, such as cat, dog, and beef proteins.^196^ The epitope cetuximab had in common with these allergens was a glycosylated sugar on the Fab region: galactose-α-1,3-galactose.^196^ To avoid such background immune responses towards PEG and certain glycans, an alternative could be to move towards chemical glycosylation of proteins, where a non-immunogenic or native glycans can be chosen to avoid background immune targeting.

As a final point, regarding future research in this field, there is a need for stronger links between therapeutic formulation, realistic stress studies and their impact on both aggregation and immunogenicity. A sizeable portion of current research that is interested in the immunogenicity of protein aggregates tend to use exaggerated stress conditions to induce their aggregates. As a result, practically every possible stress has been shown to induce immunogenic aggregates. Certain stresses may be more likely to cause immunogenic aggregates than others, but with the current approach of using exaggerated conditions, this is impossible to deduce. Improved replication of the conditions used during manufacture and processing of these proteins should be used to discover the most probable causes for aggregation. Moreover, a practical approach to investigate more representative aggregates that occur in a clinical setting would be to investigate biotherapeutics samples which have passed their shelf life, for the occurrence immunogenic aggregates. A more detailed understanding of the diverse parameters involved in the aggregation of protein therapeutics and the complex interactions between the various strategies utilized for control of protein stability, together with the immunogenic consequences of protein aggregates, is critical for the ongoing clinical development of protein therapeutics.

## Conflicts of interest

E. M. S. and P. E. C. are shareholders in Glycome Biopharma. S. F. is an employee of Glycome Biopharma.

## Supplementary Material
